# Study of genes polymorphisms in RANK/RANKL/OPG and WNT signaling pathways and their associations with bone parameters in broiler chicken

**DOI:** 10.1016/j.heliyon.2023.e22371

**Published:** 2023-11-11

**Authors:** Michala Steinerova, Cenek Horecky, Ales Knoll, Sarka Nedomova, Petr Slama, Ales Pavlik

**Affiliations:** a Department of Animal Morphology, Physiology and Genetics, Mendel University in Brno, Faculty of AgriSciences, Zemedelska 1/1665, 613 00, Brno, Czech Republic; b Department of Food Technology, Mendel University in Brno, Faculty of AgriSciences, Zemedelska 1/1665, 613 00, Brno, Czech Republic

**Keywords:** Bone, Polymorphism, Broiler, Gene, *TNFRSF11A*, *WNT1*

## Abstract

Limb problems are one of the most common problems with fast-growing meat-type chickens. Various bone abnormalities, which can lead to limping, bone weakness, or even fractures, bring overall discomfort to birds and a loss of production. Genetic aspects are often associated with these side effects on bone stability and are also cited as the dominant cause. These points to a close negative relationship of genetic selection for rapid growth with traits involved in bone integrity. Due to the assumption of an additive genetic background, improvements through genetic tools can be used. Our study is focused on selected genes of important signaling pathways for bone metabolism. We tried to detect polymorphisms that would show associations with selected bone parameters in a total of 48 broilers. Those were fast-growing Ross 308 hybrids and slow-growing Hubbard M22BxJA87A hybrids. The *TNFRSF11A* and *WISP1* genes were tested. A total of fourteen polymorphisms were found, three of them were synonymous and five in the intron. In the case of four polymorphisms found in exons of the *TNFRSF11A* gene (c.11*G* > *T*, c.31*G* > *A*, c.37*C* > *G*, c.514*G* > *A*), associations with the observed bone parameters (bone strength, bone dimensions and bone mass) were demonstrated. The genetic architecture of bone traits is not fully understood, therefore the present study and the knowledge gained can help to increase the potential in poultry breeding processes and thus reduce the death of individuals.

## 1 Introduction

Over the last few decades, a significant increase in movement problems has been detected in broilers. These are closely related to the occurrence of bone problems [10], including various deformity, bone weakness and fracture [17,26]. Among the most important factors in their formation are nutrition, environmental effect (pathogens, genetics and management practices. These also affect the final development of the bone structure. The high percentage of deformations is also caused by genetic selection, mainly due to the faster growth of broilers [34]. As compared to 1960s, their growth rate has increased by 300 % [6]. The economic benefits of faster growth are at the expense not only of bone growth and development (which cannot garner support for overgrown broiler bodies), but also of various metabolic disorders. Rapid growth and rapid weight change predispose the proximal femur prone to exertion, especially by changing posture and shifting the center of gravity [19]. All these problems have an impact on both the welfare of the broilers themselves and production, leading to economic losses. Therefore, bone disorders are also considered to be one of the main problems in poultry industry [10]. The study of the causes of bone abnormalities is necessary because many of the resulting symptoms are not clinically visible, which does not allow objective quantification of damage [34]. Also, the genetic basis and interrelationship of this issue is not fully explored and understood, which can also be said about the genetic architecture of important bone features. Thus, the present study could help to better understand the genetics of bone metabolism. This is both through the identification of new candidate genes, markers, qualification and quantification of specific bone proteins, and above all through the use of this information in practice specifically through selection in poultry breeding and increasing the potential in breeding processes.

Genetic changes, such as various structural variations in the form of single nucleotide polymorphisms (SNPs) to mutations in larger stretches, can underlie numerous diseases, not least those that cause bone fragility and frequent bone disorders. Studies with SNPs point to their importance in the variability of disease susceptibility, whereas these polymorphisms are the basis of differences between individuals [16].

The importance of RANK/RANKL/OPG and Wnt signaling pathways in bone metabolism has been demonstrated several times. Disruption of the regulation of these pathways leads to bone disorders. That is due to the important role in the differentiation of bone cells and the processes in which they are involved [35,39].

The major proteins, as the name suggests, of the RANK/RANKL/OPG signaling pathway include the receptor activator of nuclear factor-kappa B (RANK), the receptor activator of nuclear factor-kappa B ligand (RANKL), and the decoy receptor osteoprotegerin (OPG) [29]. These are important regulatory molecules of osteoclastogenesis. RANKL protein, encoded by the *TNFSF11* gene, as a differentiation and activating factor of osteoclasts, maintains and stimulates their resorption activity, thanks to its association with many hormones and cytokines. It is highly expressed in osteoblasts/stromal cells, hypertrophic chondrocytes, and primitive mesenchymal cells. Experimental administration of soluble RANKL increases osteoclast formation, which ultimately leads to osteoporosis. The RANK protein, encoded by the *TNFRSF11A* gene, exhibits very similar functions as RANKL, whereas it is equally widely expressed. OPG protein, encoded by the *TNFRSF11B* gene, which is an inhibitor of osteoclast formation and acts as a decoy receptor for RANKL, is produced by osteoblastic cells. Experiments with mice in which the gene was knockout showed the development of osteoporosis, and treatment of normal mice with OPG led to osteopetrosis. This system is important not only in the regulation, i.e., activation of bone cells and skeletal calcium management, but also in other processes, such as mammary gland physiology and epithelial proliferation during pregnancy [2, 3, 12, 20].

The various mutations occurring in the Wnt signaling pathway and the number of skeletal diseases associated with them only confirm the importance of this pathway in bone formation, development, and remodeling [28]. This signaling consists of two main pathways. The canonical pathway, which is involved in the process of osteogenesis, known as the Wnt/β-catenin pathway. The non-canonical pathway is further divided into a Wnt/Ca^2+^ dependent pathway and a Wnt/planar cell polarity (PCP) pathway [11,21]. A new candidate gene for improving bone strength and modulating osteogenesis has been identified within the Wnt signaling pathway. It is the *WISP1* gene, also known as *CCN4*, which encodes the WISP protein. Its expression has been recorded both in the case of skeletal development and in osteoblasts, or their precursors, and in bone formation or healing. In addition, studies in mice showed that individuals with knockout *WISP* showed lower bone mineral density, bone volume, and cortical bone thickness [36].

This study focused on the analysis of potential associations between selected bone mechanical parameters in hybrid combinations of broilers and SNPs, which were searched within genes involved in Wnt and RANK/RANKL/OPG signaling pathways. Based on the important role of these genes in bone metabolism, it is assumed that possible SNPs in the monitored areas affect both osteogenesis in terms of protein synthesis, activity, and production, as well as the overall formation and remodeling of bone tissue in broiler. Therefore, the study of genes that are part of bone metabolism can provide new insights leading to an understanding of the principles and basics of various bone diseases. This may be helpful in practice, through breeding processes that would reduce losses due to deaths.

## 2 Materials and methods

Hybrid combinations of fast-growing hybrids Ross 308, and slow-growing hybrid Hubbard M22BxJA87A, with a total of 48 broilers, were tested. The experiment took place in Mendel University's experimental stables, and all animals were exposed to the same conditions. The fattening of the tested broilers were carried out according to the principles stated in the technological manual for the Ross 308 hybrid combination (https://en.aviagen.com/assets/Tech_Center/Ross_Broiler/Ross-Broiler-Pocket-Guide-2020-EN.pdf), while the housing was in accordance with Council Directive 2007/43/EC on minimum rules for the protection of chickens reared for meat production. Experiments were carried out in accordance with the guidelines of The Committee of Experts for Ensuring the Welfare of Animals of the Mendel University in Brno (Reference number of accreditation 57199/2020-MZE-18134) and those corresponding to the purposes specified in § 15, §16 and § 7 of the Law no. 246/92 Coll. as amended. From the 1st to the 10th day of age, the chickens were given a commercial feed mixture BR1. From the 10th day, the chickens were given the commercial feed mixture BR2 and from the 28th day they were given the commercial feed mixture BR3. The feeding regime was *ad libitum* with the use of tube feeders and automatic feeding from drip feeders, and shavings were used as bedding. The animals were slaughtered by decapitation at the age of 35 days, and the collected blood was stabilized with heparin. Femur bones of broilers were tested with the following parameters: bone strength, weight, width, and length. Bone strength was determined using TIRATEST 27025 testing machine (TIRA Maschinenbau GmbH, Schalkau, Germany) by a three-point bending test, while their dimensions were measured using a Vernier caliper. Measurements were taken at the longest and greatest distance between the most proximal and distal points of epiphysis and the *facies caudalis* and *facies cranialis* at the fracture point. DNA were isolated (DNA Lego kit, Top-Bio, Prague, Czech Republic), and specific oligonucleotide primers were designed for the analysis of particular genes (Table 1), where sequences from Genbank (http://www.ncbi.nlm.nih.gov/genbank/) and the OLIGO v4.0 software (Molecular Biology Insights, Inc., Colorado Springs, CO, USA) were used.

**Table 1:** *TNFRSF11A* and *WISP1* primers.

Gene	Primer sequence	Product size (bp)
*TNFRSF11A* (exon 1)	Forward: 5'-AATAGGATGCCAGAAGTTTGTGAC-3'	619
	Reverse: 5'-TCCTACGTACGTGCCTGAAGAC-3'	
*TNFRSF11A* (exon 6)	Forward: 5'-TGTAGGAGCCACGACGAGC-3'	520
	Reverse: 5'- AAGGAGACACGAGGAAAGTTGG-3'	
WISP1 (exon 5)	Forward: 5'-CACACCTCTAGATCCGGACTGTAG-3'	729
	Reverse: 5'- CTGGCTGCTCTTAATGGATGG -3'	

**Legend:** TNFRSF11A – Tumor necrosis factor ligand superfamily member 11a*, WISP1 –* Wnt1-inducible signaling pathway protein-1.

The conditions for the PCR reactions were optimized (Table 2) using the ABI Veriti 96-Well thermocycler (Life Technologies, Applied Biosystems, Foster City, CA, USA).

**Table 2:** Thermal profile of PCR reaction.

Gene/Exon	Denaturation (temperature/duration)	Cyclic denaturation, annealing, elongation (temperature/duration)	Number of cycles	Final elongation and cooling (temperature/duration)
*TNFRSF11A* (exon 1)	94 °C/3 min	94 °C/60 s 55 °C/30 s 72 °C/60 s	30	72 °C/10 min 4 °C/∞
*TNFRSF11A* (exon 6)				
WISP1 (exon 5)				

**Legend:***TNFRSF11A* – Tumor necrosis factor ligand superfamily member 11a*, WISP1 –* Wnt1-inducible signaling pathway protein-1.

**Fig. 1:**
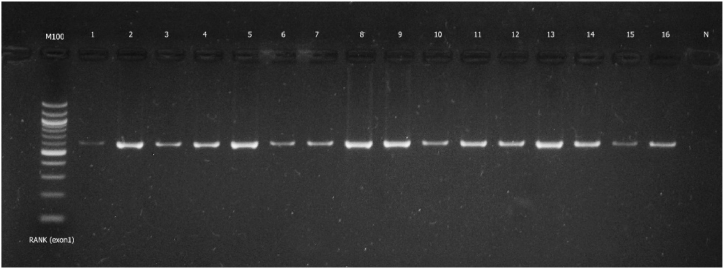
Image of agarose gel showing the size of the *TNFRSF11A* (exon 1) gene product in selected samples along with a negative control and DNA molecular weight marker (100 bp ladder). Full gel image is provided as supplementary file: “Supplementary file-Gel Image-Fig. 1”.

**Fig. 2:**
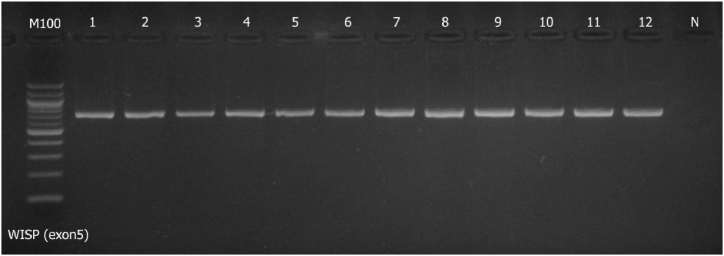
Image of agarose gel showing the size of the *WISP1* (exon 5) gene product in selected samples along with a negative control and DNA molecular weight marker (100 bp ladder). Full gel image is provided as supplementary file: “Supplementary file-Gel Image-Fig. 2”.

Amplicons were validated by 2.5 % TBE-agarose gel stained by GoodView (Ecoli, ltd., Bratislava, Slovak Republic) as shown Fig. 1, Fig. 2 in of a few representative samples. The time and voltage used for separating nucleic acid fragments were 30 min and 120 V. The preparation of PCR sequencing template is presented in Table 3).

**Table 3:** Thermal profile for PCR sequencing template.

Denaturation (temperature/duration)	Cyclic denaturation, annealing, elongation (temperature/duration)	Number of cycles	Cooling (temperature/duration)
96 °C/60 s	96 °C/10 s 50 °C/5 s 60 °C/4 min	25	4 °C/∞

PCR products were purified and subsequently the supernatant served for a sequencing reaction using BigDye XTerminator Purification kit (Life Technologies, Applied Biosystems) and an ABI PRISM 3500 DNA analyzer (Life Technologies, Applied Biosystems). For detection and analysis of mutations and identification of alleles, SeqScape v2.7 (Life Technologies, Applied Biosystems) was used.

In the case of evaluation, the non-parametric statistical Kruskal-Wallis test was used. It determines whether there is a significant difference in median values within two or more independent groups. It is more appropriate than the classical one-way analysis of variance (ANOVA) when the assumption of normality is violated. It was also used due to the small size of the tested population, for which it is also suitable due to the nature of the data, which are not normally distributed [38]. Acar and Sun [1] also emphasized its suitability for SNP testing. The genotype was chosen as the independent variable and the observed bone parameters as a dependent variable. The analysis was performed by STATISTICA 12 statistical software (StatSoft Inc., Tulsa, USA) and the overall level of statistical significance was defined as P < 0.05.

## 3 Results

Total of fourteen polymorphisms were found within selected regions of the *TNFRSF11A* and *WISP1* genes. Within the *TNFRSF11A* gene, two exons, exon 1 and 6, were studied. Furthermore, exon 5 of the *WISP1* gene was tested Of the fourteen identified polymorphisms, five were in the introns. Nine polymorphisms were in the exons, while three were synonymous and the remaining six had the potential to change the amino acid sequences.

The *TNFRSF11A* gene is located on chromosome 2 in poultry and consists of 10 exons. The PCR amplicons of testing exon 1 region were 613 bp in length and amplicons of exon 6 were 520 bp in length. Total of eight polymorphisms were found within exon 1 (c.11G > T, c.31G > A, c.37C > G, c.57C > G, c.90T > C, c.94C > G, c.96G > A, c.220 + 47C > T), of which one was in the intron (c.220 + 47C) and three were synonymous (c.57C > G, c.90T > C, c.96G > A). Of the remaining four polymorphisms (c.11G > T, c.31G > A, c.37C > G, c.94C > G) found in the exon, three showed associations with the observed parameters (bone strength and bone length) (Table 4).

**Table 4:** Association of *TNFRSF11A* (exon 1, 6) gene polymorphisms with selected bone parameters in hybrids.

Gene	SNP	Genotype (n)	Bone breaking strength [N]	Length [mm]	Width [mm]	Bone mass [g]
*TNFRSF11A* (exon 1, 6)	c.11G > T	GG (12)	148.68 ± 8.14	73.42 ± 3.17	7.37 ± 0.19	7.62 ± 0.73
	Hubbard	GT (12)	185.58 ± 11.47	72.78 ± 2.45	7.59 ± 0.23	7.92 ± 0.78
		TT (0)	0.00 ± 0.00	0.00 ± 0.00	0.00 ± 0.00	0.00 ± 0.00
	*P-value*		***0.0001***	*0.5327*	***0.0385***	*0.5102*
	c.31G > A	AA (8)	147.19 ± 8.59	74.19 ± 3.56	7.37 ± 0.11	7.69 ± 0.70
	Hubbard	GA (16)	176.44 ± 18.37	72.59 ± 2.28	7.53 ± 0.26	7.80 ± 0.80
		GG (0)	0.00 ± 0.00	0.00 ± 0.00	0.00 ± 0.00	0.00 ± 0.00
	*P-value*		***0.0034***	*0.1484*	*0.0974*	*0.8046*
	c.37C > G	CC (12)	148.68 ± 8.14	73.42 ± 3.17	7.37 ± 0.19	7.62 ± 0.73
	Hubbard	CG (12)	185.58 ± 11.47	72.78 ± 2.45	7.59 ± 0.23	7.92 ± 0.78
		GG (0)	0.00 ± 0.00	0.00 ± 0.00	0.00 ± 0.00	0.00 ± 0.00
	*P-value*		***0.0001***	*0.5327*	***0.0385***	*0.5102*
	c.94C > G	CC (0)	0.00 ± 0.00	0.00 ± 0.00	0.00 ± 0.00	0.00 ± 0.00
	Hubbard	CG (5)	151.31 ± 6.50	72.06 ± 1.63	7.37 ± 0.27	7.48 ± 0.76
		GG (19)	170.65 ± 21.43	73.33 ± 3.00	7.50 ± 0.22	7.83 ± 0.76
	*P-value*		*0.1058*	*0.3491*	*0.4956*	*0.5790*
	c.514G > A	CC (16)	168.22 ± 19.82	72.07 ± 2.35	7.47 ± 0.25	7.58 ± 0.75
	Hubbard	GC (8)	163.85 ± 22.06	75.04 ± 2.34	7.51 ± 0.19	8.29 ± 0.52
		GG (0)	0.00 ± 0.00	0.00 ± 0.00	0.00 ± 0.00	0.00 ± 0.00
	*P-value*		*0.5815*	***0.0200***	*0.6458*	***0.0428***
	c.514G > A	CC (5)	218.00 ± 35.19	76.66 ± 2.07	8.35 ± 0.39	10.50 ± 1.08
	Ross	GC (19)	250.39 ± 34.54	74.23 ± 2.85	8.23 ± 0.51	9.95 ± 0.83
		GG (0)	0.00 ± 0.00	0.00 ± 0.00	0.00 ± 0.00	0.00 ± 0.00
	*P-value*		*0.0816*	*0.1097*	*0.5221*	*0.2135*

**Legend:***TNFRSF11A* – Tumor necrosis factor ligand superfamily member 11a*, n -* numbers of individuals of each genotype, analyzed parameters listed in the table are presented as mean ± SD (Standard Deviation).

In the case of *TNFRSF11A* gene exon 6, only one polymorphism was found (c.514G > A). This polymorphism showed an association in bone length and bone mass (Table 4).

Within the studied *TNFRS11A* gene, only two genotypes were identified for each particular polymorphism. The polymorphism of c.11G > T was significantly associated with bone breaking strength between *GG* × *GT* genotypes, however, the *T* allele showed higher bone strength in the tested Hubbard hybrids than *G* allele. Another association in the same polymorphism was for the bone width parameter, where *T* allele led to wider bones than the *G* allele. The same result as for the c.11C > T polymorphism was obtained for the c.37C > G polymorphism, which, given the same allele distribution, may indicate a link between these two polymorphisms. Statistical significance in bone strength was also found for the c.31G > A polymorphism between *AA* × *GA* genotypes, where the *A* allele led to weaker bones than the *G* allele. The only polymorphism found within exon 6, c.514G > A, showed an association with bone length and mass between *CC* × *GC* genotypes and the *G* allele led to longer and heavier bones.

The *WISP1* gene, located on chromosome 2 in poultry, has 5 exons. The PCR products of the exon 5 tested region had a size of 729 bp. A total of 5 polymorphisms were found (c.1103-20C > T, c.1103-7A > G, c.1133A > G, c.1476 + 32G > C, c.1476 + 63A > G), of which four were in the intron (c.1103-20C > T, c.1103-7A > G, c.1476 + 32G > C, c.1476 + 63A > G) and one (c.1133A > G) in exon (Table 5). All three genotypes were found for all identified polymorphisms. No statistically significant difference was found for any of the found polymorphisms.

**Table 5:** Association of *WISP1* (exon 5) gene polymorphisms with selected bone parameters in hybrids.

Gene	SNP	Genotype (n)	Bone breaking strength [N]	Length [mm]	Width [mm]	Bone mass [g]
*WISP1* (exon 5)	c.1133A > G	GG (16)	167.27 ± 21.97	73.25 ± 2.28	7.43 ± 0.24	7.73 ± 0.84
	Hubbard	GA (7)	160.52 ± 12.02	72.67 ± 3.67	7.53 ± 015	7.91 ± 0.55
		AA (1)	202.35 ± 0.00	72.67 ± 0.00	7.90 ± 0.00	8.44 ± 0.00
	*P-value*		*0.2987*	*0.6880*	*0.1255*	*0.6550*
	c.1133A > G	GG (13)	236.84 ± 33.11	74.72 ± 3.42	8.29 ± 0.40	10.26 ± 1.04
	Ross	GA (10)	252.99 ± 41.54	74.69 ± 2.15	8.16 ± 0.58	9.85 ± 0.71
		AA (1)	238.52 ± 0.00	75.50 ± 0.00	8.75 ± 0.00	9.72 ± 0.00
	*P-value*		*0.4857*	*0.9555*	*0.4021*	*0.4651*

**Legend:***WISP1 –* Wnt1-inducible signaling pathway protein-1*, n -* numbers of individuals of each genotype, analyzed parameters listed in the table are presented as mean ± SD (Standard Deviation).

The range of values of individual bone parameters and their mean value was listed (Table 6).

**Table 6:** Range of values of individual bone parameters and their mean values.

Hybrid	Individuals (n)	Bone breaking strength [N]	Length [mm]	Width [mm]	Bone mass [g]
Hubbard	24	135.47–203.49	66.90–79.15	7.00–8.04	6.34–9.23
M22BxJA87A		166.76 ± 20.69	73.06 ± 2.73	7.48 ± 0.23	7.82 ± 0.76
Ross 308	24	170.52–322.21	69.48–79.88	7.57–9.22	8.71–12.61
		243.64 ± 37.08	74.74 ± 2.88	8.25 ± 0.49	10.07 ± 0.92

**Legend:***n -* numbers of individuals of each hybrid line, analyzed parameters listed in the table are presented as mean ± SD (Standard Deviation).

## 4 Discussion

The aim of this study was to find polymorphisms that would show associations with the observed bone parameters of two hybrid broiler lines. Genes of important signaling pathways that play a key role in bone metabolism were tested. There are relatively few studies that examine SNPs in relation to bone quality in animals [14,[22], [23], [24]], with studies predominating to find SNPs for different quality traits, growth rates, or production, depending on the species [4,25,37].

In this study, associations were found only in the *TNFRSF11A* gene within the Hubbard M22BxJA87A hybrids, namely in exon 1 (c.11G > T, c.31G > A, c.37C > G) and exon 6 (c.514G > A). The polymorphisms found in exon 1, c.11G > T and c.37C > G, are assumed to be in bond, through the same results. A statistically significant difference was found in the observed parameters of bone strength and bone width, with individuals with the *GG* genotype having demonstrably weaker bones, and the width of their bones was smaller than in the *GT* genotype. For the c.514G > A polymorphism, where an association was found with both bone weight and length, it could be concluded that the longer the bone, the more susceptible it may be to fractures, which has not been confirmed.

This study follows on and expands previous studies where laying hens were tested [[30], [31], [32], [33]], specifically in the study of Steinerova et al. [32], the identical genes were tested. The same C.1133A > G polymorphism was found in the *WISP* gene, exon 5, also without a significant difference. Within the *TNFRSFS11A* gene, some of the found polymorphisms were also identified (c.31G > A, c.94C > G in exon 1). In contrast to the previous study in laying hens, the c.31G > A polymorphism was associated with bone strength in the tested broiler hybrids. Horecka et al. [9] dealt with a very similar study in ISA Brown laying hybrids, in the number of 110 individuals, who tried to find polymorphisms in relation to calcium homeostasis in *ATP2B1* gene and the same observed bone parameters, whereas no significant effects were found. Jansen et al. [13] identified 16 potential candidate genes, based on their search for SNPs, that were associated with bone mineral density and bone breaking strength. Their study tested *tibiotarsus* and *humerus*. Their results on bone strength were determined by several genes, which individually have a rather small effect. Another study by Johnsson et al. [15] also on laying hens dealt with genome-wide association study of various bone parameters and body weight, in addition to comparing different housing systems. They detected three significant loci for body weight, but no significant loci for bone strength. Studies by De Koning et al. [5], Raymond et al. [27] and Guo et al. [8] also dealt with candidate genes important for bone metabolism, in connection with osteoporosis in laying hens. Specifically, Guo et al. [8] found that the 165–171 Mb region on GGA1, which is the region of the *SERPINE3*, *INTS6*, *POSTN* and *RANK* genes, has a significant effect on bone quality. The importance of these genes in bone quality can also be deduced from this. Another study in broilers and within the RANK/RANKL/OPG signaling pathway gene was reported by Grupioni et al. [7]. They searched for SNPs within the *TNFSF11* and *RUNX2* genes and associations to production traits, with one SNP found within each of them, g.14,862T > C in *TNFSF11* and g.124,883A > G in *RUNX2*, with the possibility of use in the selection to improve the characteristics of the quality of carcass and utility traits. Another comprehensive study that analyzed the genetic basis of bone traits in chickens by Li et al. [18] based on a genome-wide association experiment identified candidate genes and significant variants associated with various bone parameters in 545 individuals. Their results suggest that all the significant SNPs associated with bone parameters were found on chromosomes 1, 4 and 27 and they also recognized a total of 21 candidate genes that could interfere with bone growth and development, which brings new insights into understanding the architecture of poultry bones.

## 5 Conclusions

This study reports several SNPs, within the candidate genes of important signaling pathways for bone metabolism. Within the *TNFRSF11A* gene, polymorphisms were found that showed associations with the traits we monitored. The research could expand in future to include other important genes of bone metabolism, while it should also be applied to a larger number of tested individuals, within several populations, which is a limitation of the study. The results can be used to innovate biotechnological procedures that will lead to increasing the potential in poultry breeding processes, both broilers and laying hens regarding the quality and resistance of the skeleton in relation to technological housing systems. An increase in resistance will lead to a reduction in losses due to animal deaths and an increase in the efficiency of meat and egg production. At the same time, a general reduction in the number of reared animals required for sufficient production may be achieved, due to a lower need to raise individuals to replace those who have died, which may lead to a reduction in the environmental burden caused by this agricultural sector.

## Declaration of conflict of interest

Authors have no conflict of interest to declare.

## CRediT authorship contribution statement

**Michala Steinerova:** Investigation, Methodology, Writing – original draft. **Cenek Horecky:** Formal analysis, Investigation, Methodology. **Ales Knoll:** Formal analysis, Investigation, Methodology. **Sarka Nedomova:** Formal analysis, Investigation, Methodology. **Petr Slama:** Formal analysis, Investigation, Methodology. **Ales Pavlik:** Formal analysis, Investigation, Methodology, Writing – original draft, Writing – review & editing.

## Declaration of competing interest

The authors declare that they have no known competing financial interests or personal relationships that could have appeared to influence the work reported in this paper.
