# Supplementary material for: Study of genes polymorphisms in RANK/RANKL/OPG and WNT signaling pathways and their associations with bone parameters in broiler chicken
Source: Heliyon. 2023 Nov 11;9(11):e22371. doi: 10.1016/j.heliyon.2023.e22371 (PMC10694325; doi:10.1016/j.heliyon.2023.e22371)
Supplement: Multimedia component 2 [file mmc2.docx]

**Supplementary file-Gel Image-Figure 2**


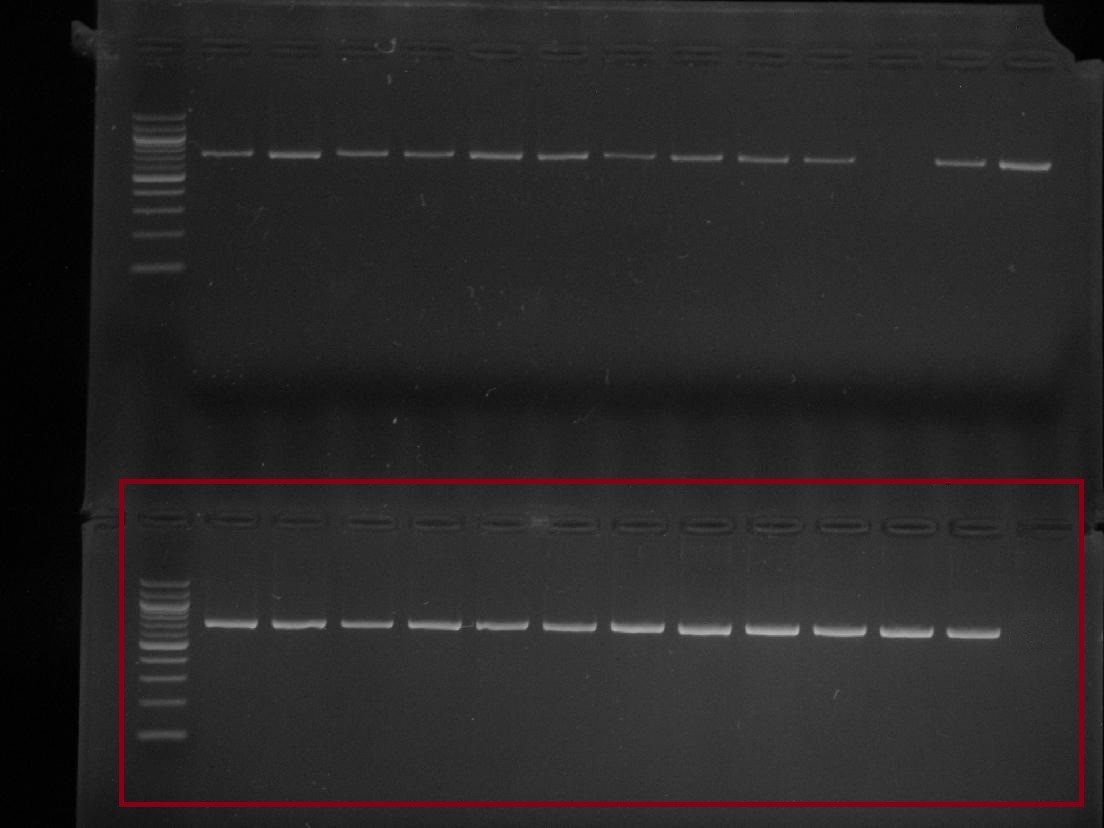


The red box corresponds to the published content in Figure 2.
